# Predicting PTSD and complex PTSD from interpersonal violence in Japanese school-based extracurricular sports activities: using the International Trauma Questionnaire (ITQ)

**DOI:** 10.3389/fpsyg.2024.1463641

**Published:** 2024-12-06

**Authors:** Hayato Toyoda, Katsuhiko Ishikawa, Yasuhiro Omi

**Affiliations:** ^1^Integrated Graduate School of Medicine, Engineering, and Agricultural Sciences, University of Yamanashi, Yamanashi, Japan; ^2^Japan Society for the Promotion of Science (JSPS), Tokyo, Japan; ^3^Graduate School of Education, Naruto University of Education, Tokushima, Japan; ^4^Graduate Faculty of Interdisciplinary Research, University of Yamanashi, Yamanashi, Japan

**Keywords:** interpersonal violence, extracurricular sports activities, post-traumatic stress disorder, complex post-traumatic stress disorder, ITQ, mental health, corporal punishment, safe sport

## Abstract

**Introduction:**

Victims of interpersonal violence in sports show various mental health concerns. However, no studies have quantitatively examined their primary complaints, considering psychological symptoms such as denial of self-concept and interpersonal challenges not captured by conventional post-traumatic stress disorder (PTSD). Recently, an association between interpersonal violence victimization and complex PTSD (CPTSD) has been noted in Japanese sports coaching situations, specifically for extracurricular sports activities. This study aimed to examine the applicability of the International Trauma Questionnaire (ITQ) and determine whether interpersonal violence victimization and related risk factors predicted PTSD and CPTSD in extracurricular sports activities in Japan.

**Methods:**

This study included 651 adults aged 18–25 who had previously participated in extracurricular sports activities in junior high and high school. The ITQ was examined using confirmatory factor analysis with maximum likelihood with robust standard errors, fit indices comparisons, a graded response model, differential item functioning, and rank correlation designs. A binomial logistic regression model with robust standard errors examined the association of PTSD and CPTSD with interpersonal violence victimization and related risk factors.

**Results:**

The optimal factor structure, measurement precision, and validity of the ITQ were confirmed. Physical and psychological violence victimization and the ITQ were positively correlated with PTSD, difficulties in emotion regulation, self-disgust, and interpersonal problems subscales, respectively. A high frequency of psychological and physical violence victimization experiences and self-identified LGB (lesbian, gay, or bisexual) were associated with PTSD and CPTSD diagnosability. Additionally, being a woman and in school life away from parents were associated solely with PTSD diagnosability.

**Discussion:**

This is the first quantitative study to examine CPTSD in a study on interpersonal violence in sports. Our findings can provide insights into desirable victim support and enhanced clinical care in interpersonal violence in a sports context.

## 1 Introduction

School-based extracurricular sports activities (hereafter, ESA) are flourishing in Japan and are recognized as a unique and excellent component of the education system (e.g., Tomozoe, 2018). Furthermore, it contributes to students' physical and mental development and realization of a satisfactory school life (e.g., Imashuku et al., 2019). Conversely, the Japanese ESA, with its unique practices and power structures, such as the excessive virtue of “never give up until the end” and control through punishment to maintain discipline with impunity, has also become a hotbed of violence (Omi, 2019). In Japanese sports, especially ESA, coach violence has a long history of being described as “corporal punishment” (Shimofure et al., 2018). Additionally, violence in sports coaching situations has recently been discussed within the physical violence of a physical nature, such as punching; non-physical psychological violence, such as threatening and discriminating, and sexual violence, such as sexual acts without consent, the framework of “interpersonal violence,” and is widely recognized internationally as a deep-rooted social problem (Mountjoy et al., 2016; Schmidt et al., 2022).

In Japan, interpersonal violence in ESA has been widely recognized since 2013, triggered by a 2012 student suicide case (Uchida et al., 2020). In response, the Japan Sport Association et al. (2013) announced the “Declaration on the Elimination of Violence in Sports.” Related organizations have also issued emergency statements, sounding an alarm in this regard (e.g., Japanese Association for Behavior Analysis, 2014). Furthermore, institutions have taken many measures, including the establishment of consultation service and the expansion of training for coaches. However, Human Rights Watch (2020), an international human rights organization, published a report, “I Was Hit So Many Times I Can't Count,” which described the serious reality of violence still being prevalent, especially in ESA. This report also highlighted the inadequate support systems for victims. Thus, the problem of interpersonal violence in sports coaching in Japan, especially in ESA environments, is attracting worldwide attention. In addition, in 2023, 10 years after the “Declaration on the Elimination of Violence in Sports,” the number of consultations on damage caused by coaches' inappropriate behavior, surveyed since 2014, reached a record high (485 cases) in 2023, which is a situation requiring immediate action (Japan Sport Association, 2024).

Until now, previous studies in Japan have focused exclusively on physical violence. Psychological violence, such as verbal abuse that deny people's dignity, have recently been included as research phenomena in Japan (Kamei and Okamoto, 2021). This has highlighted the need to clarify the psychological effects of these victimization experiences (Ohashi et al., 2022; Toyoda et al., 2023). Conversely, gradually accumulated findings, mainly from Western countries, reported that victims of interpersonal violence suffered from various mental health problems. Specifically, interpersonal violence victimization has been found to be associated with low self-esteem and post-traumatic stress disorder (PTSD; Parent et al., 2022), low quality of life in adulthood (Vertommen et al., 2018), and self-harm and eating disorders (Mountjoy et al., 2016; Willson et al., 2023). Several narrative studies also reported that they suffered from serious psychosocial disturbances, which included mood disorders, such as depression, negative self-image development, struggles with new relationships, and suicidal ideation (Kerr et al., 2020; Stirling and Kerr, 2013). In Japan, Ae (2014) argues that the trauma of experiencing violent victimization during long-term ESA led to physical disorientation toward sports, behavioral atrophy, and lack of showing initiative and assertiveness. However, few studies have quantitatively described the main complaints of victim of interpersonal violence considering the specificity of self-concept and difficulties with interpersonal problems, as suggested in previous studies (e.g., Kavanagh, 2014), and assuming various persistent and recurrent psychological symptoms.

Assuming that interpersonal violence victimization in ESA is a “difficult-to-avoid, persistent, and repetitive stress experience,” it is possible to explain the victim's core symptoms in terms of complex post-traumatic stress disorder (CPTSD), which was newly added in the 11th revision of the World Health Organization's International Classification of Diseases (ICD-11: World Health Organization, 2018). CPTSD is a concept first proposed by Herman (1992; 2015) and is based on sexual abuse. The original PTSD has been explained in terms of three symptom clusters (Re-experiencing in the here and now, Avoidance, and Sense of current threat). In contrast, CPTSD is accompanied by Disturbances in Self-Organization (DSO) in addition to the three PTSD symptom clusters described above. DSO consists of three symptom clusters: Affective dysregulation, Negative self-concept, and Disturbances in relationships. Both are not distinguished by specific event criteria as a prerequisite and are judged by symptom nature (i.e., presence or absence of DSO symptoms; Cloitre et al., 2018; Daniunaite et al., 2021). It is also characteristic that PTSD comorbidity is not diagnosed for CPTSD (Cloitre, 2020).

CPTSD has primarily been discussed in the context of childhood abuse and war damage (e.g., Brewin et al., 2017). Conversely, Harada (2020, 2021) found that not all victims of interpersonal violence developed CPTSD but inferred that brutal violence in Japanese sports (daily excessive discipline, corporal punishment, and cursing) could cause CPTSD. Indeed, interpersonal violence victimization strips athletes of their agency through a lack of ability to regulate emotions, long-term feelings of resignation and helplessness, and fosters a sense of passivity (Kavanagh, 2014). According to Kerr et al. (2020), in addition to conventional PTSD symptoms, victimization has lasting negative effects on the maintenance and breakdown of subsequent interpersonal relationships and causes negative self-concepts.

Recent studies have made extensive use of the International Trauma Questionnaire (ITQ: Cloitre et al., 2018), which was developed to comprehensively measure PTSD and CPTSD in accordance with ICD-11. The ITQ has already been validated for multiple trauma exposure samples. Furthermore, the six-factor correlated model and two-factor second-order model are considered optimal regarding factorial validity (Brewin et al., 2017; Fresno et al., 2023; Ho et al., 2019; Redican et al., 2021). Hence, Lofthouse et al. (2023) highlighted the need to explore how specific trauma types were associated with CPTSD. However, no previous studies addressed CPTSD in sports or ESA. Thus, the ITQ's reliability, validity, and factor structure for interpersonal violence victimization in sports and ESA as traumatic events also remain unclear. Therefore, they will then be necessary to examine correlations with other scales that are predicted to correspond to the ITQ subscales and to clarify whether they can be identified as symptoms for interpersonal violence victimization in ESA. By doing so, it would be possible to examine the psychological symptoms of interpersonal violence victimization using the ITQ.

This study aimed to extensively examine the applicability of the ITQ to interpersonal violence victimization in ESA in Japan and clarify the main complaints of psychological symptoms that victims of interpersonal violence have. Additionally, the few research studies in this area have shown that certain social categories, such as gender and minority group (e.g., LGB), can be risk factors for interpersonal violence, regardless of the relationship with the perpetrator (e.g., Mountjoy et al., 2016; Parent and Vaillancourt-Morel, 2021). Based on the above, risk factors related to interpersonal violence in sports (sex, sexual minority, handicap, school life away from parents, early sports specialization) and sports experience (highest sport level, sport type) noted in previous studies (Daignault et al., 2023; Mountjoy et al., 2016; Parent and Fortier, 2018; Parent and Vaillancourt-Morel, 2021; Raum et al., 2023; Vertommen et al., 2016) were also considered to explore their impact on PTSD and CPTSD simultaneously.

## 2 Materials and methods

### 2.1 Participants and procedure

A screening survey was conducted among 7,000 adults aged 18–25 years who had registered for a survey tool, “Freeasy,” provided by iBRIDGE Corporation. In the screening survey, participants were asked whether or not they had ever belonged to an ESA in junior high and high school using nominal scale level questions. We selected 2,332 participants with experience in organized ESA in throughout the 6 years of junior high and high schools. The main survey was capped at 1,200 participants due to funding constraints, and the response form was automatically closed when the response data reached 1,200. All surveys were timed for July 2023. Responses with incomplete data, such as consecutive identical responses and those who violated the Directed Questions Scale (DQS; Maniaci and Rogge, 2014), were excluded. Finally, data from 651 (*M* = 21.31, *SD* = 1.97, 208 men, 443 women) participants were analyzed of the 1,200 who responded to the main survey with consent. For details of descriptive statistics of socio-demographics, please see Table 1.

**Table 1:** Socio-demographics and sports experience of this study.

	**Men (*****n*** = **208)**		**Women (*****n*** = **443)**		**Total (*****n*** = **651)**	
	***n***	**%**	***n***	**%**	***n***	**%**
**Age**						
18 years	13	6.25	56	12.64	69	10.60
19 years	22	10.58	62	13.00	84	12.90
20 years	14	6.73	61	13.77	75	11.52
21 years	36	17.31	67	15.12	103	15.82
22 years	36	17.31	58	13.09	94	14.44
23 years	44	21.15	75	16.93	119	18.28
24 years	42	20.19	63	14.22	105	16.13
25 years	1	0.48	1	0.23	2	0.31
**Sexual orientation**						
Heterosexual	191	91.83	371	83.75	562	86.33
Homosexual (gay, lesbian)	5	2.40	3	0.68	8	1.23
Bisexual	3	1.44	19	4.29	22	3.38
Other	1	0.48	12	2.71	13	2.00
N/A	8	3.85	38	8.58	46	7.07
**Handicap due to any chronic illness or disability**						
Yes	17	8.17	43	9.71	60	9.22
No	180	86.54	369	83.30	549	84.33
N/A	11	5.29	31	7.00	42	6.45
**Sports type in junior high school**						
Individual sport	93	44.71	224	50.56	317	48.69
Team sport	115	55.29	219	49.44	334	51.31
**Sports type in high school**						
Individual sport	85	40.87	197	44.47	282	43.32
Team sport	123	59.14	246	55.53	369	56.68
**Highest sports level in junior high and high school**						
Municipal	65	31.25	147	33.18	212	32.57
Prefectural	74	35.58	126	28.44	200	30.72
Regional	31	14.90	59	13.32	90	13.83
National	17	8.17	59	13.32	76	11.67
Best 4 or above national (include international)	3	1.44	19	4.29	22	3.38
Not participant in any tournament	18	8.65	33	7.45	51	7.83
**School life away from parents**						
Had experienced	28	13.46	44	9.93	72	11.06
Had not experienced	180	86.54	399	90.07	579	88.94
**Early sports specialization**						
Had experienced	76	36.54	140	31.60	216	33.18
Had not experienced	132	63.46	303	68.40	435	66.82

N/A, not answer; School life from away parents = experience of living in a dormitory away from parents to lead an extracurricular sports activities centered life in junior high school and high school; Early sport specialization = experience of training exclusively and intensively in a single sport for at least 8 months per year (basically all year round), starting before the age of 12.

### 2.2 Measures

#### 2.2.1 Japanese version of interpersonal violence in sport

The IViS-J (Toyoda et al., 2023) was used to measure the frequency of experiences of interpersonal violence victimization from coaches in junior high and high school ESA. This scale was developed by translating items from the original scale of Vertommen et al. (2016) and uses a different scoring method than the original scale. An example of an item was the following psychological violence included “You were shouted or cursed at,” while physical violence included “You were slapped/hit with an open hand.” It consists of 18 items with two factors. Responses were rated on a 5-point Likert scale that ranges from “*Never*” to “*Always*.” The ω coefficient was adequate (psychological violence = 0.885; physical violence = 0.906).

#### 2.2.2 International Trauma Questionnaire

To measure PTSD and CPTSD for interpersonal violence victimization as measured by the IViS-J, we used Cloitre et al.'s (2018) ITQ, which was developed based on the ICD-11. It consists of 18 items, and responses were rated on a 5-point Likert scale that ranges from “*Not at all*” to “*Extremely*.” This comprehensive and simple instrument measures three PTSD symptoms (1. re-experiencing in the here and now, 2. avoidance, and 3. sense of current threat), PTSD functional impairment (PTSDFI), three DSO symptoms (1. affective dysregulation, 2. negative self-concept, and 3. disturbances in relationships), and DSO functional impairment (DSOFI). Three PTSD symptoms and three DSO symptoms were measured by two items each. An example of an item was the following re-experiencing in the here and now included “Having upsetting dreams that replay part of the experience or are clearly related to the experience?” avoidance included “Avoiding internal reminders of the experience (for example, thoughts, feelings, or physical sensations)?” sense of current threat included “Being “super-alert,” watchful, or on guard?” affective dysregulation included “When I am upset, it takes me a long time to calm down,” negative self-concept included “I feel like a failure,” and disturbances in relationships included “I feel distant or cut off from people.” PTSDFI and DSOFI were discriminated by the scores of responses to each of the three items (e.g., Affected your relationships or social life?). In addition to the scoring scale scores for each symptom, the ITQ was also compatible with the calculation of diagnostic scores according to the ICD-11 diagnostic algorithm (Cloitre et al., 2018). A score of ≥ “*Moderately*” indicated symptom endorsement. A Japanese translation was published by the International Trauma Consortium (2017) and its guidelines were used in this study. The traumatic event for the ITQ was “experience of interpersonal violence victimization during ESA in junior high and high school,” which was the response given in the IViS-J. This was operationally explained in the instructional text during the ITQ response. The ITQ is designed to measure symptoms in the past month (International Trauma Consortium, 2017), so the same instructions were used in this study. The ω coefficient was adequate (re-experiencing in the here and now = 0.835, avoidance = 0.905, sense of current threat = 0.857, PTSD = 0.925, affective dysregulation = 0.825, negative self-concept = 0.932, disturbances in relationships = 0.858, and DSO = 0.927).

#### 2.2.3 Impact of event scale-revised

We used the IES-R developed by Asukai et al. (2002) as an index that corresponded to the three symptoms of PTSD on the ITQ. It consists of 22 items with three factors. Responses were rated on a 5-point Likert scale that ranges from “*Not at all*” to “*Extremely*.” The ω coefficient was adequate (intrusion = 0.953, avoidance = 0.952, and hyperarousal = 0.934).

#### 2.2.4 Japanese version of the difficulties in emotion regulation scale

We used the J-DERS, developed by Yamada and Sugie (2013) as an index that corresponds to “affective dysregulation” in the DSO of the ITQ. It consists of 16 items with four factors. Responses were rated on a 5-point Likert scale that ranges from “*Almost never*” to “*Almost always*.” The ω coefficient was adequate (non-acceptance of emotional responses = 0.935, behavior control difficulties = 0.917, and limited access to emotion regulation strategies = 0.925), except for lack of emotional awareness (0.342, inadequate).

#### 2.2.5 Self-disgust scale

We used the SDS, developed by Mizuma (1996) as an index that corresponds to “negative self-concept” in the DSO of the ITQ. It consists of 21 items with single factor. Responses were tracked on a 5-point Likert scale that ranges from “*Almost never*” to “*Almost always*.” The ω coefficient was adequate (self-disgust = 0.984).

#### 2.2.6 Japanese short version of the inventory of interpersonal problems (IIP-32)

We used the IIP-32 developed by Suzuki et al. (2022) as an index that corresponds to “disturbances in relationships” in the DSO of the ITQ. It consists of 32 items with eight factors. Responses were rated on a 5-point Likert scale that ranges from “*Not at all*” to “*Very much*.” The ω coefficient was adequate (dominant = 0.912, competitive = 0.894, cold = 0.907, socially inhibited = 0.919, non-assertive = 0.927, exploitable = 0.876, dependent = 0.898, and intrusive = 0.882).

For all scale details on the descriptive statistics, normality test, and internal consistency, please see Supplementary material 1.

### 2.3 Data analysis

**Figure 1:**
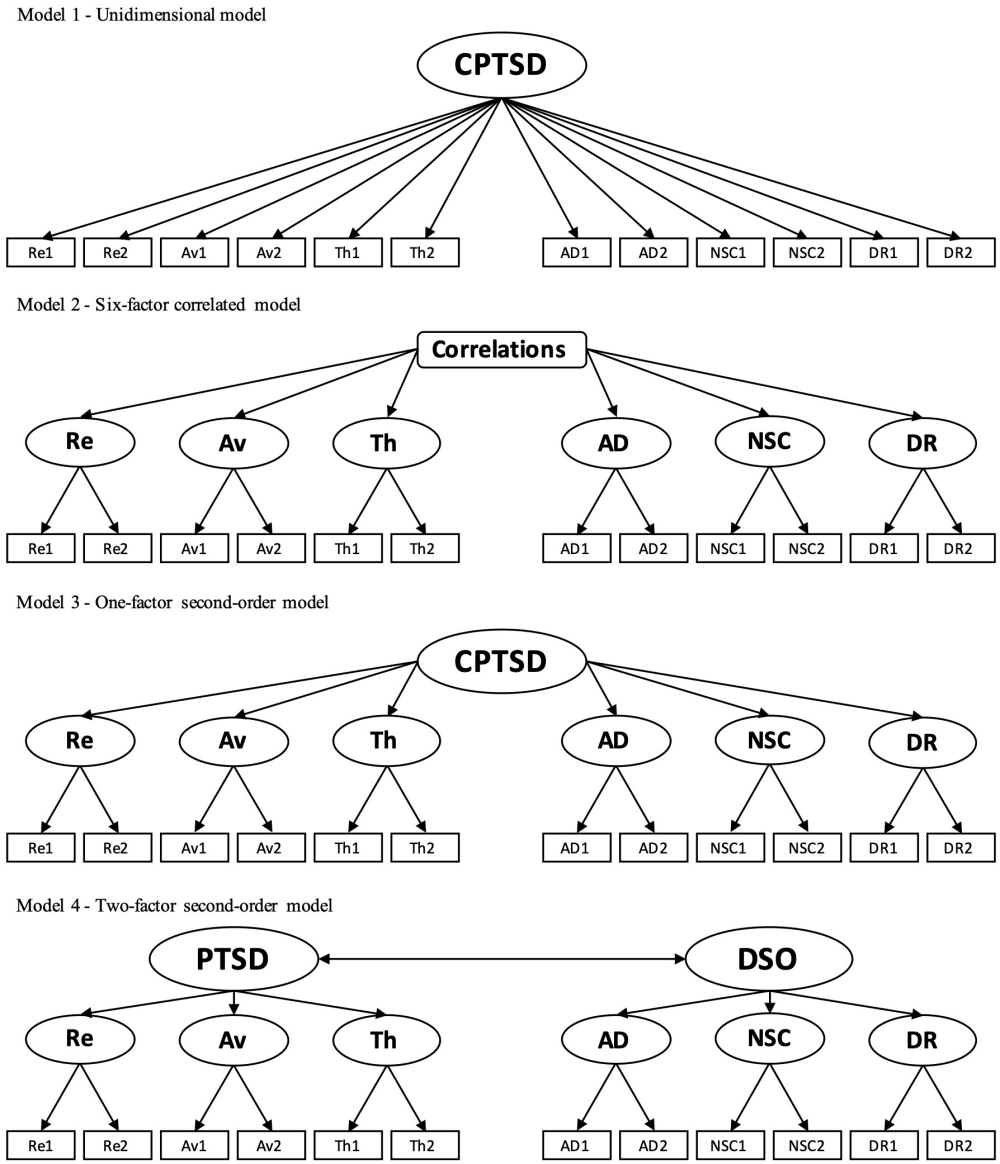
Candidate optimal models for confirmatory factor analysis.

Internal consistency was confirmed by the ω coefficient. A Shapiro-Wilk test was used to assess the presence of a normal distribution. To estimate the ITQ's latent structure, we established a candidate optimal model considering previous studies (e.g., Redican et al., 2021). Specifically, we established four models (Figure 1): unidimensional (Model 1), six-factor correlated (Model 2), one-factor second-order (Model 3), and two-factor second-order (Model 4). These models were assessed via confirmatory factor analysis (CFA) and model comparisons by fit indices. The maximum likelihood with robust standard errors (MLR) was employed for parameter estimation. MLR helps compute robust estimates independent of the multivariate normal distribution assumption (Li, 2016). The fit indexes used were Chi-square value (χ^2^), Comparative Fit Index (CFI), Root Mean Square Error of Approximation (RMSEA), Tucker-Lewis Index (TLI), Standardized Root Mean Square Residual (SRMR), Akaike Information Criterion (AIC), Bayesian Information Criterion (BIC). The criteria were CFI and TLI ≥0.95 and ≥0.90, RMSEA ≤ 0.06 and ≤ 0.10, and SRMR ≤ 0.08 and ≤ 0.10 for good and acceptable, respectively (Hu and Bentler, 1999; Kline, 2005). The AIC and BIC were relative indices in the multiple models, with smaller values that approximated the optimal model (Kubo, 2022). There were no assumptions of residual covariance among the observed variables in all the models.

Categorical factor analysis with weighted least squares (WLS) method was performed for each of the PTSD and DSO subscales to confirm uni-dimensionality. Subsequently, the Graded Response Model (GRM; Samejima, 1969) of the Item Response Theory and Differential Item Functioning (DIF; Thissen et al., 1993) based on the likelihood-ratio test, were applied to evaluate the ITQ's item characteristics (item discrimination *a* and item difficulty *b*) and equivalence by attribute (non-uniform and uniform DIF). The target group for the DIF analysis was sex (men and women). The association between the scale mean scores was examined by Spearman's rank correlation coefficient (ρ). The criteria to determine the correlation coefficient was ±0.10–0.30, ±0.40–0.60, and ±0.70–0.90 for a weak, moderate, and strong effect, respectively (Dancey and Reidy, 2011).

We generated categorical variables for PTSD and CPTSD as diagnostic possibilities in accordance with the ITQ diagnostic scoring protocol (Cloitre et al., 2018; International Trauma Consortium, 2017). Specifically, those with all three PTSD symptoms and functional impairment were identified as “Diagnosability of PTSD.” Those with all six symptoms of PTSD (three each of PTSD and DSO) and functional impairment were identified as “Diagnosability of CPTSD.” Additionally, a binomial logistic regression analysis (robust standard errors, forced entry method) was conducted via psychological and physical violence victimization of the IViS-J and related risk factors as independent variables and PTSD and CPTSD diagnosability as dependent variables. The criterion for the occurrence of multicollinearity was Variance Inflation Factor (VIF) ≥5 (Shrestha, 2020). The significance level was set at 5%. JASP version 0.18.1 and jamovi version 2.4.8 were used for all the statistical analyses.

### 2.4 Ethical considerations

This study was approved by the Ethical Review Board of Faculty of Education, University of Yamanashi (No. R5-017). The survey participants were provided with the consent information beforehand, including questions about their negative experiences. The study was conducted with due attention to informed consent, and we made preparations and considerations for the survey being voluntary and ensured that it was possible to forcibly close the browser even in the middle of a response, arranged for any psychological support, such as a counselor, if necessary, by contacting the authors via the contact information provided in the survey form as appropriate. Regarding the process of conducting the survey, the response data were unsigned and converted into a statistically processed, non-personally identifiable ID. The data used for the analysis was limited to the data of completed response transmissions in which consent to cooperate in the survey was obtained. There were no requests for psychological support by the survey participants after the survey was completed.

## 3 Results

### 3.1 Factor structure and item-level assessment of the ITQ

A CFA was used to examine the factor structure of the ITQ by comparing the models (Table 2). Consequently, Model 2 (six-factor correlated) and 4 (two-factor second-order) were accepted as interpretable (see Figure 1 for each model). The relative evaluation was slightly better for Model 2 than Model 4; however, the difference in the BIC values was small (< 10).

**Table 2:** Model fit statistics with confirmatory factor analysis of the ITQ.

	**χ^2^**	**df**	***p***	**CFI**	**TLI**	**RMSEA [90%CI]**	**SRMR**	**AIC**	**BIC**
Model 1	1,616.69	54	< 0.001	0.762	0.709	0.211 [0.20, 0.22]	0.090	18,593.47	18,700.95
Model 2	111.22	39	< 0.001	0.989	0.981	0.053 [0.04, 0.07]	0.021	17,118.00	17,292.66
Model 3	535.61	48	< 0.001	0.926	0.898	0.125 [0.12, 0.14]	0.071	17,524.38	17,658.74
Model 4	172.03	47	< 0.001	0.981	0.973	0.064 [0.05, 0.07]	0.041	17,162.80	17,301.63

Model 1 = unidimensional model; Model 2 = six-factor correlated model; Model 3 = one-factor second-order model; Model 4 = two-factor second-order model.

CFI, comparative fit index; TLI, tucker-lewis index; RMSEA, root mean square error of approximation; CI, confidence interval; SRMR, standardized root mean square residual; AIC, akaike information criterion; BIC, bayesian information criterion.

Confirmatory factor analysis (CFA) was estimated by the maximum likelihood method with robust standard errors (MLR).

The GRM and sex differences DIF were used to examine the ITQ's measurement precision and item-level ratings (Table 3). The uni-dimensionality of PTSD and DSO was confirmed and met the theoretical assumptions. In the GRM, two items of “negative self-concept” showed higher item discrimination than the other two symptoms. Furthermore, item difficulty was stable for all the items. No significant sex difference items were found between the groups for both non-uniform DIF and uniform DIF.

**Table 3:** Measurement precision by GRM and DIF of the ITQ items.

	**λ^a^**	**GRM**					**Non-uniform DIF**			**Uniform DIF**		
		***a***	***b*_1_**	***b*_2_**	***b*_3_**	***b*_4_**	**χ^2^**	***p***	**Adj.*p***	**χ^2^**	***p***	**Adj.*p***
**PTSD**												
Re item 1	0.877	1.82	0.66	1.40	1.90	2.46	1.75	0.186	0.372	0.06	0.804	0.964
Re item 2	0.897	2.03	0.62	1.21	1.72	2.41	1.93	0.165	0.372	1.85	0.174	0.521
Av item 1	0.949	3.02	0.62	1.20	1.65	2.13	0.06	0.802	0.869	0.00	0.985	0.985
Av item 2	0.930	2.53	0.59	1.19	1.61	2.12	1.89	0.169	0.372	0.83	0.362	0.718
Th item 1	0.900	2.07	0.46	1.06	1.50	2.08	0.78	0.377	0.566	4.14	0.042	0.251
Th item 2	0.877	1.82	0.34	0.94	1.43	2.06	0.03	0.869	0.869	0.50	0.478	0.718
**DSO**												
AD item 1	0.832	1.50	−0.24	0.76	1.38	2.06	0.07	0.787	0.787	1.46	0.227	0.340
AD item 2	0.839	1.54	0.38	1.09	1.58	2.13	0.59	0.444	0.771	3.26	0.071	0.213
NSC item 1	0.949	3.01	−0.13	0.50	1.00	1.43	0.85	0.356	0.771	1.58	0.208	0.340
NSC item 2	0.951	3.08	−0.09	0.54	1.02	1.43	1.33	0.250	0.771	0.00	0.994	0.994
DR item 1	0.867	1.74	−0.06	0.61	1.19	1.81	0.12	0.731	0.787	0.04	0.836	0.994
DR item 2	0.885	1.90	−0.11	0.48	1.02	1.61	0.43	0.514	0.771	4.75	0.029	0.176

Re, re-experiencing in the here and now; Av, avoidance; Th, sense of current threat; AD, affective dysregulation; NSC, negative self-concept; DR, disturbances in relationships.

λ^a^, factor loadings for uni-demensionality by categorical factor analysis of weighted least squares estimation. GRM, graded response model; DIF, differential item functioning based on likelihood ratio test. *a*, item discrimination; *b*, item difficulty; Adj.p, *p*-value adjusted by the Benjamini-Hochberg procedure with likelihood ratio test using multiple comparisons.

### 3.2 Correlation between interpersonal violence victimization, the ITQ, and each psychological symptom

The association between the mean scores of each scale was examined via the correlation coefficients (Table 4). Psychological and physical violence showed weak-to-moderate positive correlations with the six symptoms of the ITQ and with PTSD and DSO. Similarly, it also revealed weak-to-moderate positive correlations with psychological symptoms that corresponded to the six symptoms of the ITQ. The six symptoms were also moderately to strongly positively correlated with their corresponding psychological symptoms. Regarding the ITQ and other psychological symptoms, the correlation coefficients were relatively higher for psychological violence than for physical violence.

**Table 4:** Correlations of interpersonal violence victimization, the ITQ, and related psychological symptoms.

	**IViS-J**		**ITQ-PTSD**				**ITQ-DSO**			
	**PS**	**PH**	**Re**	**Av**	**Th**	**PTSD**	**AD**	**NSC**	**DR**	**DSO**
**IViS-J**										
Psychological violence victimization (PS)	–	0.51	0.49	0.47	0.53	0.56	0.51	0.51	0.50	0.57
Physical violence victimization (PH)	0.51	–	0.38	0.34	0.38	0.39	0.29	0.29	0.30	0.32
**IES-R**										
Intrusion	0.52	0.37	0.67	0.65	0.62	0.70	0.62	0.58	0.63	0.67
Avoidance	0.49	0.33	0.63	0.65	0.63	0.69	0.60	0.57	0.61	0.65
Hyperarousal	0.51	0.35	0.62	0.63	0.66	0.70	0.64	0.61	0.66	0.70
**J-DERS**										
Non-acceptance of emotional responses	0.39	0.27	0.42	0.45	0.51	0.49	0.59	0.58	0.62	0.65
Behavior control difficulties	0.38	0.23	0.41	0.44	0.48	0.48	0.58	0.55	0.61	0.63
Limited access to emotion regulation strategies	0.40	0.26	0.44	0.46	0.52	0.51	0.61	0.59	0.63	0.67
**SDS**										
Self-disgust	0.43	0.26	0.45	0.46	0.50	0.53	0.56	0.71	0.65	0.71
**IIP-32**										
Dominant	0.35	0.27	0.38	0.40	0.38	0.41	0.45	0.40	0.47	0.49
Competitive	0.35	0.24	0.41	0.45	0.46	0.48	0.49	0.49	0.54	0.56
Cold	0.38	0.29	0.42	0.44	0.48	0.48	0.53	0.57	0.62	0.62
Socially inhibited	0.39	0.29	0.46	0.46	0.52	0.51	0.52	0.58	0.63	0.64
Non-assertive	0.39	0.29	0.44	0.47	0.50	0.50	0.52	0.58	0.61	0.63
Exploitable	0.38	0.28	0.42	0.47	0.48	0.49	0.54	0.58	0.60	0.64
Dependent	0.36	0.29	0.44	0.43	0.47	0.48	0.51	0.50	0.53	0.56
Intrusive	0.34	0.26	0.40	0.42	0.42	0.44	0.47	0.44	0.49	0.52

Re, re-experiencing in the here and now; Av, avoidance; Th, sense of current threat; AD, affective dysregulation; NSC, negative self-concept; DR, disturbances in relationships.

Statistics in this table are Spearman's rank correlation coefficient (ρ). All correlation coefficients were significant at *p* < 0.001.

### 3.3 Associations between PTSD and CPTSD with interpersonal violence victimization and related risk factors

Among participants (*n* = 651), 7.68% (*n* = 50) and 6.30% (*n* = 41) met the criteria for PTSD and CPTSD, respectively, as per the ITQ diagnosis. After each diagnosability group was coded as reference class 1, we verified whether interpersonal violence victimization and related factors predicted diagnosability via a binomial logistic regression analysis (Table 5). No multicollinearity was observed in either model (VIF < 1.588), which indicated adequate model fit and prediction accuracy.

**Table 5:** Associations between PTSD and CPTSD with interpersonal violence victimization and related risk factors.

	***B***	***Robust SE***	***Wald***	***p***	***OR* [95%CI]**
**Diagnosability of PTSD**					
Psychological violence victimization	1.18	0.26	18.31	< 0.001	3.26 [0.67, 1.69]
Physical violence victimization	0.56	0.25	3.86	0.025	1.74 [0.07, 1.04]
Sex (women)^a^	1.07	0.51	3.81	0.037	2.91 [0.06, 2.07]
Sexual orientation (LGB self-identification)^b^	1.53	0.65	5.29	0.019	4.61 [0.25, 2.80]
Handicap due to any chronic illness or disability (yes)^c^	0.47	0.61	0.66	0.433	1.61 [−0.71, 1.66]
Sports type in junior high school (team sport)^d^	0.43	0.62	0.21	0.691	1.28 [−0.97, 1.46]
Sports type in high school (team sport)^e^	0.11	0.56	0.04	0.851	1.11 [−1.00, 1.21]
Sports level in junior high and high school (above national)^f^	0.53	0.55	1.06	0.340	1.70 [−0.56, 1.61]
School life away from parents (had experienced)^g^	1.68	0.52	10.97	0.001	5.37 [0.67, 2.69]
Early sports specialization (had experienced)^h^	−0.11	0.49	0.06	0.818	0.89 [−1.07, 0.85]
**Diagnosability of CPTSD**					
Psychological violence victimization	1.21	0.27	16.16	< 0.001	3.37 [0.69, 1.74]
Physical violence victimization	0.51	0.25	2.98	0.039	1.67 [0.03, 1.00]
Sex (women)^a^	0.77	0.55	1.75	0.160	2.15 [−0.30, 1.83]
Sexual orientation (LGB self-identification)^b^	1.91	0.64	8.26	0.003	6.78 [0.66, 3.17]
Handicap due to any chronic illness or disability (yes)^c^	0.68	0.62	1.22	0.271	1.97 [−0.53, 1.89]
Sports type in junior high school (team sport)^d^	0.49	0.67	0.69	0.464	1.63 [−0.82, 1.79]
Sports type in high school (team sport)^e^	−0.20	0.60	0.13	0.738	0.82 [−1.37, 0.97]
Sports level in junior high and high school (above national)^f^	0.79	0.57	2.10	0.168	2.20 [−0.33, 1.91]
School life away from parents (had experienced)^g^	1.10	0.61	3.53	0.071	2.99 [−0.10, 2.29]
Early sports specialization (had experienced)^h^	−0.48	0.56	0.83	0.387	0.62 [−1.58, 0.61]

Base *n* = 651. Forced entry method was used for variable imputation. Degrees of freedom (df)s = 1. *B*, partial regression coefficient; *Robust SE*, robust standard error; *Wald*, statistic to check the significance of the estimated partial regression coefficients; OR, odds ratio; CI, confidence interval. VIF = 1.058–1.588. Valuation index and predictive accuracy in the model with diagnosability of PTSD as dependent variable: $\chi_{\left(519\right)}^{2}$ = 89.721, *p* < 0.001; Nagelkerke *R*^2^ = 0.411; Deviance = 162.813 (vs. null model = 252.534); Accuracy = 0.951; Area of under the curve (AUC) = 0.908; Specificity = 0.992. Valuation index and predictive accuracy in the model with diagnosability of CPTSD as dependent variable: $\chi_{\left(519\right)}^{2}$ = 72.716, *p* < 0.001; Nagelkerke *R*^2^ = 0.387; Deviance = 140.645 (vs. null model = 213.361); Accuracy = 0.960; AUC = 0.885; Specificity = 0.996.

The respective REFs showed: ^a^men; ^b^heterosexuals who did not self-identify as LGB; ^c^no; ^d, e^individual sport; ^f^low sports level including municipal, prefectural, and regional; ^g, h^had not experienced.

When PTSD was the dependent variable, five factors independently and significantly promoted diagnosability: high frequency of psychological and physical violence victimization, women (vs. men), LGB self-identification (vs. heterosexuals who did not self-identify as LGB), and school life away from parents (vs. had not experienced). Additionally, when CPTSD was the dependent variable, three factors independently and significantly promoted diagnosability: psychological and physical violence victimization and LGB self-identification (vs. heterosexuals who did not self-identify as LGB).

## 4 Discussion

This study demonstrated that the ITQ was fully applicable to interpersonal violence victimization in ESA in Japan (Tables 2–4). Moreover, we found that a high frequency of psychological and physical violence victimization experiences and self-identified LGB were associated with PTSD and CPTSD diagnoses (Table 5). Conversely, being a woman and in school life away from parents was solely associated with a PTSD diagnosis (Table 5). Our findings extend previous studies (e.g., Omi and Fujine, 2021; Parent et al., 2022) that showed an association between interpersonal violence in sports and mental health problems. This was the first quantitative study to examine both PTSD and CPTSD aspects in sports and the ESA context.

### 4.1 Factor structure, measurement precision, and validity of the ITQ

The factor structure of the ITQ for specific trauma types measured in this study was appropriate for a six-factor correlation (Model 2) and two-factor second-order models (Model 4). This aligned with representative systematic reviews of the ITQ (Brewin et al., 2017; Redican et al., 2021). The BICs of Models 2 and 4 remained below 10, indicating that both models' validity could be explained (Raftery, 1995). Regarding the ITQ item-level measurement precision examined by the GRM, all items were high and stable. Typically, item discrimination is “high” at 1.35–1.69 and “very high” at 1.70 or higher (Baker, 2001). Additionally, disqualification criteria are item discrimination and difficulty of 0.5 or lower and 4.0 or higher in absolute value, respectively (Toyoda, 2002). Therefore, the ITQ was judged as a relatively unbiased measure with an appropriate range width and consistent with the latent trait. Although previous studies that express the psychological characteristics of the ITQ via IRT and DIF remain scarce, low item discrimination was detected in an item of the DSO factor in a German general population sample (Christen et al., 2021). Furthermore, sex differences in the DIF were found in an item of the PTSD factor in a clinical refugee sample (Nielsen et al., 2023). Owing to the differences in the different trauma types targeted, precise comparisons between these studies were difficult. However, since no sex differences in the DIF were observed in our results, there may not be differences in response tendencies between men and women derived from the ITQ items, at least in the ESA in Japan.

For the rank correlations, we also found that significant positive correlations between each scale were found that confirmed the construct validity of the ITQ measured for interpersonal violence victimization. Specifically, PTSD symptoms on the ITQ were strongly positively correlated with the subscales of the IES-R, an existing PTSD scale, at 0.6 or higher, respectively. In addition, DSO symptoms on the ITQ showed moderate to strong positive correlations with the existing J-DERS, SDS, and IIP-32 subscales corresponding to DSO symptoms, ranging from 0.4 to 0.7, respectively. Hence, the results emphasize the harmfulness of interpersonal violence victimization in ESA and its association with various psychological symptoms at both the individual and relational levels.

### 4.2 Impact of interpersonal violence victimization

In this study, < 10% of the total sample had a possible diagnosis of PTSD and CPTSD with functional impairment, which was expected in this non-clinical sample. Most participants who had a diagnostic potential for PTSD met the criteria for diagnostic potential for CPTSD, which was important, albeit at the level of descriptive statistics.

The frequency of psychological and physical violence victimization experiences was substantially associated with PTSD and CPTSD diagnosability. This result indicated that this was not just the effect on monogenic PTSD (Parent et al., 2022; Toyoda et al., 2023). In other words, we found that interpersonal violence in sports had a serious impact on interpersonal difficulties, avoiding interactions with others, struggling to establish new relationships (Kerr et al., 2020), the development of feelings of self-worthlessness, and long-term mood disturbances (Kavanagh, 2014; Stirling and Kerr, 2008) such as negative psychopathology closely related to cognition and emotion. This reflected the complexity of CPTSD, which encompassed the main symptoms of PTSD and was accompanied by DSO symptoms. This supported the trial proposal (Harada, 2020, 2021) regarding the possible expression of CPTSD in Japanese sports settings. According to Harada (2020), individuals with CPTSD (i.e., victims) in sports situations form traumatic memories, resulting in intra- and interindividual psychological and physical disorders. In addition, such traumatic memories can be activated by certain triggers, resulting in a mode change from a reassuring mode to a hostile and confusing mode, which further aggravates the victim's unstable state (Harada, 2020). This finding is generally consistent with that of other studies on childhood abuse (Cloitre et al., 2006; Nishizawa, 1997), in which survivors lacked agency and experienced serious chronic problems in interpersonal relationships that cannot be explained by the conventional concept of PTSD. According to Herman (2015), a climate of violent domination in an abusive environment is accompanied by an overwhelming sense of isolation and alert hyperarousal. In Japanese ESA, the environment in which corporal punishment occurs is also recognized as a closed space with strong tension wherein common sense does not prevail (Muramoto and Matsuo, 2016). Therefore, the similarity in both these environments made the application of findings from childhood abuse notable and complementary to our discussion.

### 4.3 Risk factors predicting diagnosability of PTSD

We found that “being a woman” and “in school life away from parents” were identified as risk factors that promoted PTSD diagnosability. Regarding sex, generally, girls are more likely to be victims of psychological violence and neglect than boys (Parent and Vaillancourt-Morel, 2021). Parent et al. (2022) reported a significant association between psychological violence, neglect victimization, and low self-esteem solely among girls. Hence, biological and psychosocial factors specific to women may be latent in the pathological structure of PTSD caused by interpersonal violence victimization in sports. Incidentally, the higher risk of developing PTSD in women than in men (Asukai et al., 2002; Olff et al., 2007) is a generalized finding that complemented our explanation.

Regarding school life away from parents, the risk of distancing oneself from parents and guardians and engaging in sports was noted among elite athletes (Parent and Fortier, 2018). Daignault et al. (2023) identified that the tendency to play sports away from their parents was a characteristic of people who had experienced repeated psychological violence victimization from multiple perpetrators and had mental health concerns. Our analysis did not consider the specific length of time that the participants spent away from their parents during their 6 years of junior high and high school. However, ESA in Japan is often dominated by a strong discipline of hierarchical structures and relationships between coaches and club members. Furthermore, the culture of perpetuating and concealing violence is possible (Ishimura and Tasato, 2017; Omi, 2019). Additionally, dormitory life in such a cultural context tends to form an even more closed environment (Omi, 2019). Victims of interpersonal violence could be exposed to an environment in which these psychosocial and material resources were depleted (school life prioritized ESA in which violence was prevalent), thereby contributing to PTSD at the diagnostic level. Studies on childhood abuse indicate that the most severe effects of traumatic events are more likely to occur in those who had limited resources for support prior to the event, which also makes recovery from the trauma difficult (Cloitre et al., 2006).

### 4.4 Risk factors predicting diagnosability of CPTSD

We found that “being LGB self-identification” was identified as a risk factor that promoted CPTSD diagnosability. In sports, LGBT and asexual individuals reportedly experience higher rates of interpersonal violence victimization (e.g., Zogg et al., 2024). Additionally, in school physical education and sports settings in Japan, many sexual minority students experience forced gender roles and are subjected to violence and ridicule owing to gender identity (Kazama et al., 2011).

Previous meta-analyses robustly supported that people who identified as LGBTQ were more prone to PTSD than those who did not (Marchi et al., 2023). A recent study examined the association between LGBTQ and CPTSD and found that exposure to gender expression-related harassment in minority stress is positively correlated with all PTSD and DSO symptoms (Charak et al., 2023). Furthermore, CPTSD has a mediating effect on traumatic events and lifetime suicide attempts (Ellis et al., 2023). However, these known arguments were not necessarily generalizable to sexual minority populations in sports violence. Conversely, the latest literature on athlete abuse reported that LGBTQ athletes are prone to lower wellbeing and self-harm (Willson et al., 2023). Thus, our new finding that LGB is a potential risk factor for harm and also a strong predictor of psychiatric diagnostic trends for CPTSD is an extension of previous research.

However, this study did not consider the extent to which our data includes violent acts or discriminatory remarks directly related to sexual orientation and identity. Hence, the above interpretation should be considered. Moreover, those who have gender dysphoria or are sexual minorities might not necessarily experienced trauma due to “extreme threat or fear” as indicated by the ICD-11; instead, they may have developed CPTSD due to the accumulation of a significant amount of daily exposure to trivial microaggressions (Harima, 2021). Additionally, participation in ESA in Japan shapes students' stereotypical gender consciousness and reinforces the power and role structures between men and women (Mikami, 2022). In Japan these days, there is often an ostensible mood in which homosexuality is welcomed, but the main factors in favor of homosexuality are the changing consciousness of the international community and the tendency toward irrelevance to oneself, pointing to the existence of so-called “apparent attitudes” (e.g., Motoyama, 2022). Given this background of Japanese society, it is possible that a sense of latent discrimination and prejudice may remain in the context of ESA in terms of interpersonal violence in ESA against LGB. Therefore, CPTSD caused by interpersonal violence may be more clearly explained by clarifying the nature of the specific type of violence directed at LGB groups and surrounding environmental factors.

### 4.5 Limitations and future directions

Considering this was a self-reported cross-sectional survey, it was difficult to refer to causal relationships or clinical assessments and recall bias of experiences may be an issue. A longitudinal study that considers the duration of exposure and a combinate study with clinical interviews by experts is required. Our sample was limited to young adults; hence, future studies should replicate our findings with a wider age sample.

As our findings were explained in the specific context of ESA in Japan, cultural factors may have influenced the results. Furthermore, this study design did not consider sports instruction situations other than ESA in middle and high schools (e.g., local sports team). Therefore, it is difficult to generalize our findings to the entirety of sports in Japan. However, since ESA is a reasonable condensation of Japanese culture (Omi, 2019) and represents Japanese sports situations where problems of interpersonal violence are persistent (Human Rights Watch, 2020), our findings are meaningful. Future studies should explore the impact of trauma types other than interpersonal violence. Peer bullying and interpersonal violence from parents are important targets (Parent et al., 2023; Vertommen et al., 2022). Especially in Japan, the promotion of violence by parents has become a social problem, as evidenced by the coined term “sport-toxic parents.” This has attracted attention due to parents' covering up of violence by coaches and excessively pushing children to exceed in competition (Shimazawa, 2022).

Our study did not include sexual violence victimization. Previous studies on interpersonal violence in sports in Western countries have examined the adverse effects of sexual violence victimization (Parent et al., 2019, 2022). However, the unreported rate of child sexual abuse is very high in Japan. Additionally, bias regarding self-reporting is likely to occur due to hesitation to report due to shame or threats from the perpetrator (Takaoka, 2016). Even in sports, many disincentives for self-reporting exist for victims of sexual violence (Human Rights Watch, 2020). Thus, sexual violence in Japan has a particular inherent measurement problem, especially when compared to other forms of violence. Future studies should carefully examine the psychiatric effects of sexual violence victimization, while controlling for social desirability.

Finally, as a more practical future direction, this study may also have implications for how victim support should be provided. For example, in the context of childhood abuse in Japan, there are already treatments that have been shown to be effective in improving CPTSD through clinical trials (Skills Training in Affective and Interpersonal Regulation Narrative Therapy; Niwa et al., 2022). Based on the results of the present study, it may be necessary to build on these clinical findings and further investigate treatments specific to the context of sports and ESA.

## 5 Conclusion

Limited studies have investigated the association between interpersonal violence in sports and mental health problems worldwide (Schmidt et al., 2022; Willson et al., 2023). In addition, the ESA examined in this study are the most representative scenes of sports activities in Japan and are essentially positioned as extracurricular activities conducted through the voluntary and spontaneous participation of students. However, ESA in Japan are rather anti-democratic and unreasonable in nature, and have become some kind of evil “sanctuary” that is difficult for the educational system to reach (Omi, 2019; Uchida, 2023). This study found that interpersonal violence victimization and several associated risk factors predicted the diagnosability of PTSD and CPTSD. This study was the first to use CPTSD in a study on interpersonal violence in sports.

From a practical perspective, in current sports contexts, athlete-standard educational interventions on interpersonal violence and risk factors are required (Zogg et al., 2024). Furthermore, just “introducing” protective policies for victims is insufficient; the “effect” of these policies should be examined longitudinally (Hartill et al., 2023). Our results indicate “the beginning of a desirable direction for the protection of human rights” toward the development and enhancement of victim support and clinical care systems specific to sports and ESA in Japanese context.

## Data Availability

The raw data supporting the conclusions of this article will be made available by the authors, without undue reservation.
