# Supplementary material for: Predicting PTSD and complex PTSD from interpersonal violence in Japanese school-based extracurricular sports activities: using the International Trauma Questionnaire (ITQ)
Source: Front Psychol. 2024 Dec 6;15:1463641. doi: 10.3389/fpsyg.2024.1463641 (PMC11659647; doi:10.3389/fpsyg.2024.1463641)
Supplement: Supplementary file 1 [file Table_1.DOCX]

Supplementary Material

**Supplemental material 1. Descriptive statistics, normality, and internal consistency.**

|  | Mean | SD | Median | Skewness | Kurtosis | Shapiro-Wilk test | | McDonald’s ω |
| --- | --- | --- | --- | --- | --- | --- | --- | --- |
|  |  |  |  |  |  | *W* | *p* |  |
| IViS-J  Psychological violence victimization  Physical violence victimization  ITQ  Re-experiencing in the here and now  Avoidance  Sense of current threat  PTSD  Affective dysregulation  Negative self-concept  Disturbances in relationships  DSO  IES-R  Intrusion  Avoidance  Hyperarousal  J-DERS  Nonacceptance of emotional responses  Behavior control difficulties  Limited access to emotion regulation strategies  SDS  Self-disgust  IIP-32  Dominant  Competitive  Cold  Socially inhibited  Non-assertive  Exploitable  Dependent  Intrusive | 1.85  1.22  1.48  1.50  1.68  1.55  1.85  2.11  2.08  2.01  1.63  1.76  1.74  1.84  2.00  1.92  2.12  1.50  1.59  1.75  1.93  1.94  1.88  1.73  1.58 | 0.82  0.59  0.84  0.91  1.02  0.84  1.02  1.26  1.19  1.05  0.92  1.02  1.02  1.02  1.08  1.09  1.13  0.83  0.88  1.01  1.09  1.10  1.02  0.98  0.88 | 1.55  1.00  1.00  1.00  1.00  1.17  1.50  2.00  2.00  1.67  1.13  1.13  1.17  1.50  1.75  1.50  1.76  1.00  1.25  1.25  1.50  1.50  1.50  1.25  1.25 | 1.36  3.96  1.97  2.06  1.54  1.85  1.35  0.98  0.92  1.04  1.64  1.31  1.45  1.34  1.06  1.13  1.00  2.11  1.81  1.52  1.14  1.13  1.17  1.54  1.80 | 1.69  17.52  3.42  3.79  1.58  3.10  1.24  -0.21  -0.19  0.32  2.00  0.77  1.25  1.13  0.24  0.31  -0.04  4.25  2.88  1.56  0.38  0.34  0.49  1.79  2.81 | 0.866  0.429  0.644  0.623  0.715  0.712  0.803  0.817  0.838  0.865  0.726  0.765  0.756  0.806  0.850  0.816  0.863  0.665  0.718  0.760  0.822  0.822  0.827  0.765  0.711 | <.001  <.001  <.001  <.001  <.001  <.001  <.001  <.001  <.001  <.001  <.001  <.001  <.001  <.001  <.001  <.001  <.001  <.001  <.001  <.001  <.001  <.001  <.001  <.001  <.001 | .885  .906  .835  .905  .857  .925  .825  .932  .858  .927  .953  .952  .934  .935  .917  .925  .984  .912  .894  .907  .919  .927  .876  .898  .882 |

*Note*. Lack of emotional awareness, a subscale of the J-DERS, was excluded because no internal consistency was found.
